# Physiological indicators of stress and meat and carcass characteristics in tail bitten slaughter pigs

**DOI:** 10.1186/1751-0147-55-75

**Published:** 2013-10-30

**Authors:** Anna Valros, Camilla Munsterhjelm, Eero Puolanne, Marita Ruusunen, Mari Heinonen, Olli A T Peltoniemi, A Reeta Pösö

**Affiliations:** 1 Research Centre for Animal Welfare, Department of Production Animal Medicine, Faculty of Veterinary Medicine, University of Helsinki, P.O.Box 57, 00014 Helsinki, Finland; 2 Department of Food and Environmental Sciences, Faculty of Agriculture and Forestry, University of Helsinki, P.O. Box 66, 00014 Helsinki, Finland; 3 Saari Unit, Department of Production Animal Medicine, Faculty of Veterinary Medicine, University of Helsinki, Pohjoinen pikatie 800, 04920 Saarentaus, Finland; 4 Reeta Pösö, Department of Veterinary Biosciences, University of Helsinki, P.O. Box 66, 00014 Helsinki, Finland

**Keywords:** Tail biting, Pig, Cortisol, HSP70, Meat quality, Carcass characteristics

## Abstract

**Background:**

Tail biting is a common welfare problem in pig production and in addition to being a sign of underlying welfare problems, tail biting reduces welfare in itself. The aim of this study was to evaluate the effects of tail biting on different *pre* and *post mortem* indicators of stress in slaughter pigs and on carcass and meat characteristics. A total of 12 tail bitten (TB) and 13 control (C) pigs from a farm with a long-term tail biting problem were selected for salivary cortisol analyses before and after transport to the slaughterhouse. After stunning, samples were taken for the analysis of serum cortisol, blood lactate, intestinal heat shock protein 70 (HSP70), and meat quality characteristics. In addition, body temperature immediately after and muscle temperature 35 min after stunning were measured, as well as lean meat percentage and carcass weight.

**Results:**

TB pigs showed a lower cortisol response to the transport-induced stress than C pigs and also had a lower serum cortisol concentration after stunning. HSP70 content in the small intestine was higher in the TB pigs than in C pigs. TB pigs had a considerably lower carcass weight therefore produced a lower total amount of lean meat per carcass than C pigs.

**Conclusions:**

This study suggests that prolonged or repeated stress in the form of tail biting causes a blunted stress response, possibly a sign of hypocortisolism. In addition, it underlines the importance of reducing tail biting, both from an animal welfare and an economic point-of-view.

## Background

Tail biting is a common and serious welfare problem in pig production. In countries where tail docking is prohibited, the prevalence of tail damage in slaughter pigs has been reported to be as high as 6-11.7% [1,2] and around 3% in countries where tail docking is allowed [3].

In addition to being a sign of underlying welfare problems, tail biting as such reduces welfare. Tail bitten pigs are more prone to get infections, such as abscesses and arthritis [1,4]. Tail damage due to biting may also have adverse effects on carcass characteristics, as it may reduce growth [4,5] and cause an increase in condemnations at slaughter [1,5]. Reduced welfare, at least in the form of a barren environment, increases the risk for tail biting [6,7] and can also have negative consequences on meat quality [8]. Even though there are many reasons to suppose that tail biting is linked to underlying stress [9,10] and that being a victim is stressful [11], there is still scarce information available on the consequences of tail biting to the victim.

The evaluation of prolonged or repeated, i.e. chronic, psychological stress is challenging, and to get a reliable picture several measures should be used [12]. Cortisol is a traditional measure of stress in pigs, being elevated by acute stress [13]. The effects of chronic stress on cortisol concentrations, however, are less straightforward. Studies on humans and laboratory animals show that chronic stress or pain appears to ultimately cause a reduction in daily overall cortisol secretion, as well as in cortisol reactivity to stressors (for a review, see [14]). Similar results have been reported in pigs housed in barren environments or under repeated noise stress [15-17].

Premortem stress affects muscle pH *post mortem*. The formation of DFD (Dark, Firm, Dry meat) is associated with long-lasting pre-slaughter stress, e.g. during handling, transport and slaughterhouse lairage as well as a long fasting time [18-20]. In the DFD case, the glycogen reservoirs are diminished already before slaughter, due to the stress-induced degradation of muscle glycogen. Therefore, the ultimate lactic acid is lower than normally resulting in a pH value higher than 6.0. In the PSE (Pale, Soft, Exudative meat) case, those pigs that still have a normal glycogen level at slaughter and that have experienced psychological and/or physical stress just before slaughter, have a fastened muscle glycogen breakdown perimortem. Lactic acid accumulates in the muscle when the muscle temperature is still high, and this combination causes a partial denaturation of meat proteins and thus a light colour and softness as well as a decrease of water-holding capacity.

Heat shock proteins (HSPs) are a potential measure of chronic stress. Cells react to stress by synthesizing HSPs, which help them to maintain intracellular protein homeostasis. HSP-induction is caused by several different cell-level stressors [21]. Among the stress-inducible HSPs, the response of HSP70 has been studied most extensively. Its synthesis peaks 8-10 hours after stress, and the concentration stays high for several days [21]. Therefore, short to intermediate transport and pre-slaughter handling may not last long enough to have an effect on the amount of HSP70 and it has been speculated to reflect stressors the pigs have encountered on the farm [22].

The aim of this study was to evaluate the effects of tail damage due to tail biting on different *pre* and *post mortem* indicators of stress in slaughter pigs and on carcass and meat characteristics by comparing pigs with tail damage to control pigs with clinically healthy tails. We hypothesised that tail damage causes stress to the victim, which will be reflected in a change in cortisol secretion during a stressful situation, i.e. transport, in an increase in HSP70 and in reduced meat quality, due to changes in muscle metabolism during the pre-slaughter handling. We report several signs of increased chronic stress in tail bitten pigs.

## Methods

### Animals and general housing conditions

Altogether 25 pigs from a farm that had had frequent problems with tail biting were selected on the farm before transport to the slaughterhouse in the morning. As this was, to our knowledge, the first study of this type, the number of pigs was decided mainly from a practical point-of-view. It was estimated that this was the maximum number of pigs we could sample during one slaughtering session.

The farm used all in–all out management by room and housed 8-9 pigs in similar standard-sized pens. One fourth of the pen floor consisted of concrete slats and the rest was solid concrete. The farmer gave the pigs a very small amount of sawdust as enrichment. The pigs were fed liquid feed from a long through, from which all pigs were able to eat at the same time. Water was delivered for the pigs freely from one water nipple in each pen.

Two veterinarians selected 12 case pigs with clearly visible tail wounds (TB) and 13 control pigs from pens where no tail biting occurred (C). They inspected the pigs and ear tagged them individually. The TB pigs had bitten tails; otherwise the TB and C animals were clinically free from signs of disease. All pigs came from one room of about 800 finishing pigs at approximately 100-115 kg live weight. Pigs for the TB and C groups were selected evenly within the room and both groups included 8 castrates, the rest of the animals being gilts. Within each pen one or two pigs had visible tail wounds. Selection of C pigs occurred in pens with no tail biting and aimed at a similar gender distribution as in TB pigs. Consequently, the pigs came from a total of 22 different pens (11 pens with tail biting, 11 control pens), with one or two pigs from each pen. All pigs had arrived at the finishing farm at the same time (at around 25 kg live weight) and were thus of approximately the same age.

Transport to the slaughterhouse started at 07:00 h, after an approximately 30-min loading period, and lasted one hour. Before transport, the pigs had been fasted since the previous afternoon. TB and C pigs were kept in separate pens during transport. At the slaughterhouse the pigs were randomly divided into two stunning groups and TB and C pigs were mixed. Stunning of Group 1 began at 09:00 h (6 TB and 6 C pigs) and stunning of Group 2 at 12:00 h (6 TB and 7 C pigs). Pigs were stunned with carbon dioxide in groups of 3 and driving to stunning was mainly automatic.

An ethical approval for the study was obtained from the Ethics Board of Viikki Campus of the University of Helsinki.

### Saliva and blood sampling and assays

Two saliva cortisol samples per pig were collected: one basal sample in the home pens prior to clinical inspection (between 06:00 and 07:00 h), before the normal feeding time of the pigs, and a post-transport saliva sample after arrival and mixing at the slaughterhouse (between 08:00 and 09:00). Saliva samples were taken using Salivette® tubes (Sarstedt AG & Co, Germany), allowing each animal to chew on a cotton swab for approximately one minute. The swab was then replaced in the tube, transported on ice in a cool box to the laboratory within 10 hours, and centrifuged at 3000 rpm for 10 minutes. The saliva was frozen at-18°C until analysis. The salivary cortisol concentration was analysed by radioimmunoassay (Coat-A-Count Cortisol, Orion Diagnostica, Turku, Finland) with modifications for use with pig saliva [23]. The method has been validated for use with pig saliva [24].

Blood samples were collected during bleeding directly after stunning for assessment of cortisol and lactate. For lactate analysis blood was collected in tubes that contained sodium fluoride, and for cortisol in serum tubes. Tubes were kept on ice during transport to the laboratory. The samples were analysed on the following day using an automatic lactate analyzer (YSI 2300 STAT, Yellow Springs Instrument Co., Yellow Springs, OH, USA). For cortisol analyses serum was separated by centrifugation in the laboratory and kept frozen (-70°C) until analysed. Serum cortisol concentrations were measured by radioimmunoassay (Coat-A-Count Cortisol, Diagnostic Products Corporation, Los Angeles, CA, USA).

### Heat shock protein70

For the analysis of HSP70 tissue samples were taken from the stomach, small intestine (approximately 1 m from the *pylorus*), large intestine *(proximal colon)* and the semimembranosus muscle *(M. semimembranosus)* within 1.5 hours after stunning. Samples taken from the gastrointestinal tract were rinsed free of luminal contents in physiological saline, cut into pieces and frozen in liquid nitrogen. The muscle samples were blotted dry and frozen in liquid nitrogen. All the samples were kept at-70°C until analysis. The inducible HSP70 was analyzed using a HSP70 EIA kit (EKS-700, StressGen Biotechnologies Corp, Victoria, Canada) which has been tested by the producer and found to recognize porcine HSP70. Tissues were homogenized with FastPrep™ homogenizer (FP120, Bio101, ThermoSavant, Savant Instruments, Holbrook, NY, USA) in the buffer provided in the kit for protein extraction and supplemented with protease inhibitor cocktail (P8340, Sigma, St. Louis, MO, USA). A tissue piece of 250 mg was homogenized in 500 μL of buffer with three times 40 s homogenization.

### Carcass and meat characteristics

The slaughterhouse provided the data about carcass weight and lean meat percentage. The latter was measured with a Hennessy Grading probe (Hennessy Grading Probe GP4, Hennessy Grading Systems, Auckland, New Zealand). Body temperature was measured from rectum 1-2 minutes after stunning (T_rectum_,°C) and muscle temperature at 5 cm depth from the semimembranosus muscle 35 min after stunning (T_35min_ ,°C). The pH value was measured from the semimembranosus muscle 35 minutes *post mortem* (pH_35min_) by homogenizing one gram of muscle sample in 10 ml of 5 mM Na-iodoacetate + 150 mM KCl, and by measuring the pH of the homogenate at room temperature. The ultimate pH was measured directly from the semimembranosus muscle (pH_24h_) 24 h *post mortem*. A combined glass electrode was used (Knick Portamess 752 pH-meter with Mettler Toledo Inlab 427 electrode).

### Muscle glycogen, lactate and glycolytic potential

Glycogen concentration was determined from the semimembranosus muscle according to [25]. Ten μl of homogenate was hydrolyzed in 200 μl of 1 M HCl at 100°C for 2 h, after which pH was adjusted to 6.5-7.5 and glucose was determined via NADP^+^ reduction with linked assay involving hexokinase and glucose-6-phosphate dehydrogenase (Glucose (HK) 16-50, Sigma Diagnostics). Lactate concentration was determined from the homogenate via NAD^+^ reduction with a linked assay involving lactate dehydrogenase and glutamate pyruvate transaminase (Boehringer-Mannheim no. 139 084). Glycolytic potential was calculated as follows [26]: GP (mmol lactate/kg) = [2*(Glycogen + Glucose + G-6-P) + (Lactate)].

### Meat colour and drip loss

Meat colour was measured from the semimembranosus muscle 24 hours *post mortem* with a Minolta Chromameter CR-200, (Minolta Camera Co. Japan) set at D_65_ illuminant after blooming for 5 min. Samples were taken from the middle of the muscle. The lightness (L*) and redness (a*) values were recorded from the average of three readings across each muscle surface. Drip loss was determined [27] by weighing muscle samples of 100 g that were then put in a plastic bag, stored for two days at 4°C and reweighed. The drip was the weight difference, expressed as percentage. Two replicate samples were determined on each muscle sample.

### Statistical analyses

The change in cortisol due to stress during loading, transport and mixing at the slaughterhouse was estimated as the difference between the home pen and the post-transport cortisol concentration. Due to technical and practical problems, one of the pre-transport (C pig) and four (one C pig and 3 TB pigs) of the post-transport saliva samples were missing from the data.

All measures related to chronic stress (saliva and serum cortisol, blood lactate, HSP70 and body temperature) and carcass (carcass weight, lean meat percentage) and meat quality (glycogen and lactate content and glycolytic potential of the semimembranosus muscle, meat colour, drip loss) were normally distributed. Therefore, the effect of tail damage on the different measures was tested using ANOVA. In addition to the test variable (TB vs C) which was added as a fixed factor, also stunning group was forced into the models as a fixed factor. In addition, the ANOVA model for lean meat percentage included carcass weight as a covariate. Preliminary analyses showed that gender did not influence any of the measures, and gender was thus not included in the models. Interaction terms for test variable × stunning group only contributed significantly (*P* < 0.05) when testing for the effect of TB on serum cortisol and stomach HSP, and were not included in any of the other final models. The appropriateness of each model was considered by examining the normality of the residuals.

The difference between cortisol concentration in the home pen and post-transport in TB and C pigs was evaluated using Paired Sample t-tests.

A significant effect was reported when *P* < 0.05. Numeric results are given as estimated marginal means and standard error (SE).

All statistical analyses were performed with the SPSS for Windows (version 12.0.1) software.

## Results

### Saliva and serum cortisol and blood lactate

**Figure 1:**
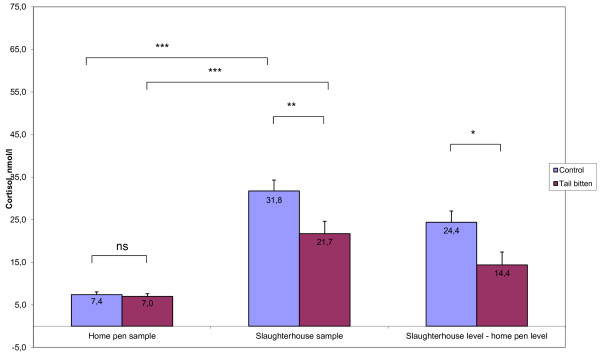
Mean (SE) cortisol levels in saliva from tail bitten (N = 12) and control pigs (N = 13), sampled in the home pen and in slaughterhouse, and the difference between these two values (Paired Sample t-test, *** *P* < 0.001; ** *P* < 0.01; * *P* **< 0.05; ns *P*** **> 0.1).** One home pen sample (C pig) and four slaughterhouse samples are missing (one C pig, three TB pigs). Means are given as data labels.

**Figure 2:**
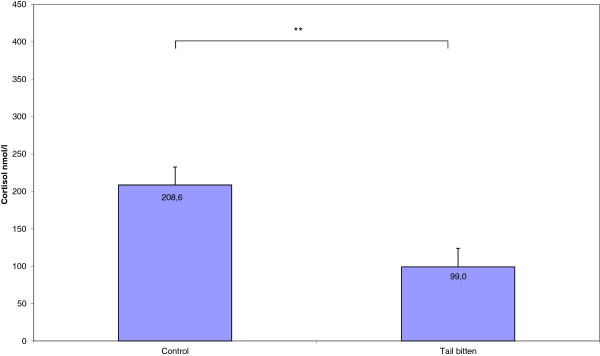
Mean (SE) cortisol levels in blood from tail bitten (N = 12) and control (N = 13) pigs, sampled at bleeding directly after stunning (Paired Sample t-test, ** *P* **< 0.01).** Means are given as data labels.

The concentration of cortisol in saliva increased during loading and transport in both C (*P* < 0.001) and TB (*P* < 0.001) pigs (Figure 1). In TB pigs the post-transport concentrations of cortisol in saliva and serum (at exsanguination) were lower (*P* = 0.02 and *P* = 0.005, respectively) and the transport-induced change in cortisol concentration was lower (*P* = 0.02) than in C pigs. The home-pen concentration of cortisol did not differ between C and TB pigs (*P* = 0.72) (Figures 1 and 2). Blood lactate concentrations did not differ between C (3.80 ± 0.40 mmol/l) and TB (4.58 ± 0.42 mmol/l) pigs (*P* = 0.19).

### HSP70

Concentrations of HSP70 differed between TB and C pigs only in the small intestine, being higher in TB pigs than in C pigs (*P* < 0.05) (Table 1).

**Table 1:** HSP70 values in control (N = 13) and tail bitten (N = 12) pigs

**HSP70**	**Control**	**Tail bitten**	**Significance**
Large intestine, ng/g tissue	1446 ± 281	1554 ± 292	0.79
Small intestine, ng/g tissue	2151 ± 263	2949 ± 273	0.047
Stomach, ng/g tissue	421 ± 76	370 ± 79	0.64
Semimembraneosus muscle, ng/g tissue	183 ± 41	253 ± 43	0.26

Results are given as estimated marginal means and standard error.

### Carcass and meat characteristics

Data on carcass and meat characteristics are presented in Table 2. The carcass weight was lower (*P* < 0.001) and lean meat percentage higher (*P* = 0.003) in TB pigs than in C pigs. The body temperature measured after stunning tended to be higher in the TB pigs than in C pigs (*P* = 0.09). There were differences neither in glycogen and lactate content nor in glycolytic potential of the semimembranosus muscle between TB and C pigs. The meat was lighter in TB pigs than C pigs (*P* = 0.002) but there were no differences in drip loss and meat redness between the two groups (*P* > 0.1). No pigs in this study had muscle pH_24h_ –values above 6.0 or pH_35min_ below 5.8 indicating no cases of DFD or PSE meat.

**Table 2:** Comparison of carcass and meat characteristics between control (N = 13) and tail bitten (N = 12) pigs

	**Control**	**Tail bitten**	**Significance**
Carcass weight, kg	82.4 ± 2.0	67.7 ± 2.1	< 0.001
Meat-%^a^	56.2 ± 0.60	60.8 ± 0.48	0.003
T_rectum_,°C ^b^	38.9 ± 0.12	39.2 ± 0.13	0.09
T_35min_ ,°C ^c^	39.8 ± 0.16	40.2 ± 0.16	0.13
pH_35min_^d^	6.73 ± 0.03	6.75 ± 0.03	0.57
pH_24_^e^	5.56 ± 0.03	5.57 ± 0.03	0.81
Glycogen (mmol/kg)	58.2 ± 4.0	62.8 ± 4.2	0.44
Lactate (mmol/kg)	46.1 ± 3.0	43.7 ± 3.1	0.59
Glycolytic potential (mmol/kg) ^f^	162.5 ± 6.8	169.2 ± 7.1	0.50
Drip loss,%	6.30 ± 0.56	6.79 ± 0.58	0.56
L* (lightness)	46.0 ± 0.57	48.9 ± 0.59	0.002
a* (redness)	8.49 ± 0.30	8.00 ± 0.31	0.26

Results are given as estimated marginal means and standard error.

^a^Meat-%: Lean meat percentage measured with Hennessy device; ^b^T_rectum_,°C: body temperature measured from rectum after stunning; ^c^T_35min_ ,°C: temperature measured from the semimembraneosus muscle 35 min after stunning; ^d^pH_35min_: pH value measured from the semimembraneosus muscle 35 min after stunning; ^e^pH_24h_: pH value measured from the semimembraneosus muscle 24 hours after stunning; ^f^Glycolytic potential (mmol lactate/kg): [2* (Glycogen + Glucose + G-6-P)] + (Lactate)].

## Discussion

We found evidence that tail damage is associated with changes in the stress physiology and carcass characteristics of slaughter pigs. Even though this is based on a limited number of animals from only one farm, the finding is reasonable as it can be assumed that tail biting is associated with stress in the pigs. In conjunction with the current study we also found that tail damage caused severe infections, possibly causing long-lasting pain [28]. Pain is a well-known cause of stress. In addition to causing pain, being a tail biting victim can be stressful in other ways, e.g. as victims are often chased and might avoid feeding at the trough in fear of exposing the tail (for a review, see [10]).

Our finding that tail bitten pigs had a significantly lower concentration of saliva cortisol after transport than non-bitten control pigs may seem contradictory to earlier results [29], showing elevated cortisol levels in tail bitten pigs, measured from single saliva samples taken in the home pen. This might be due to the fact that most of the tail bitten animals in the earlier study [29] had signs of mild fresh biting, while most of the pigs in our study had been bitten severely and chronically, as evident from our earlier published results [28]. Human and rodent studies have shown that traumatic events and long-lasting or repeated stress can cause a blunted day-rhythm in cortisol. This phenomenon is called hypocortisolism [14] and can also be seen as a reduced increase in cortisol secretion when faced with acute stressors [14] or in the ACTH test [30]. In our study, the lower cortisol post-transport in both saliva and serum in tail bitten pigs as compared to control pigs was probably due to a decreased cortisol secretion during transport, rather than to a blunted day-rhythm, as there was no difference between bitten and control pigs in the cortisol samples taken in their home pen before transportation. Even though serum and saliva cortisol cannot be directly compared, the fact that cortisol, as measured from both these media, was increased in tail bitten pigs compared to control pigs strengthens our results. The difference in cortisol, when comparing saliva and serum is at an expected level and the responses should be comparable, as there is a high correlation between saliva and serum levels, and the timing of the response peak is very similar [31].

Histopathological analyses from the same pigs, published elsewhere, revealed signs of inflammation and an increased acute phase protein response [28]. There was also a higher level of carcass condemnations in tail bitten pigs, especially due to abscesses, which suggests that the tail damage was already chronic. This has implications for the results of the current study, as tail bitten pigs might have suffered from infections, causing changes in their cortisol secretion. The interaction between the immune system and stress is complicated and studies of cortisol during disease challenges have shown both an increase and a decrease in cortisol response following disease challenge [32-34].

The stress-induced increase in the amount of HSP70 is a result from gene activation followed by synthesis of mRNA and the protein [35]. This cascade takes time; in porcine intestine the increases at the protein level are seen approximately 6 hours after the insult [35]. The time course of HSP70 induction together with the present finding of elevated HSP70 concentrations in the tail bitten pigs support the view that the tail bitten pigs had experienced an increased level of stress already prior to transportation. As the exact timing for the stress-induced rise in HSP is not known, we included stunning group in the statistical model evaluating the effect of tail damage on pig HSP. The pigs slaughtered in the later stunning group had experienced approximately 5 hours of antemortem handling, which is close to the 6 hour estimation mentioned above. However, in the current study the HSP values were actually numerically lower (data not shown) in pigs in the second stunning group, indicating that this was not the case.

While the current study design does not allow for a conclusion on cause and effect regarding tail damage and body weight, the difference between tail bitten and control pigs in carcass weights supports previous results on a negative effect of being tail bitten on growth [5,36]. In TB pigs meat percentage was higher but due to the large difference in carcass weight the amount of lean meat was considerably smaller than in C pigs (41.2 vs 46.3 kg). Even though we do not have information on the exact date when tail damage first occurred, previously published results from the same pigs used here [28] suggest that the tail damage was chronic, which might help explain the large difference in carcass weight. Chronic pain or discomfort has been suggested to retard growth in tail-bitten pigs [4], possibly partly due to reduced feed intake. Chronically bitten pigs have been reported to be reluctant to spend time at a trough feeder, as this exposes the tail [10]. In addition, stress, in this case caused by pain or by being bitten as such, may have a negative effect on the food conversion efficiency [37]. It cannot be excluded, that there was a common underlying illness causing both the victimisation and at least partly the reduced growth. Recent studies have shown a two-way connection between illness and tail biting. Pigs with e.g. leg problems have a higher risk of becoming tail bitten [38]. The animals in our study did not show clinical signs of illness, but that does not exclude the possibility of subclinical symptoms, or an earlier period of poor health.

Only minor differences in meat characteristics were found in the current study. Stress increases body temperature [39] and high muscle temperature immediately *post mortem* weakens meat quality as seen as increasing drip and in lighter meat colour [39]. The temperature in the semimembranosus muscle was not significantly higher in tail bitten than in control pigs 35 minutes post mortem in the current study. However, the meat was lighter, and the body temperature measured from rectum after stunning was higher in tail bitten than in control pigs, further supporting the finding that tail bitten pigs had experienced a higher level of stress.

## Conclusions

Tail bitten pigs showed a lower cortisol increase after transport and ante-mortem handling than non-bitten control pigs. This indicates a less reactive HPA-axis, possibly due to prolonged or repeated stress in their home pen, due to pain and to the biting as such. Results from HSP70 support these results. This study also indicates that HSP70 is a promising measure for chronic stress, while the cortisol response during acute stress is not an unambiguous reflection of previously experienced stress level. The study showed support for the fact that tail bitten pigs might produce less lean meat per carcass.

## Competing interests

The authors declare that they have no competing interests.

## Authors’ contributions

All authors were included in the planning of the study design and sampling procedures, as well as in data collection and preparing the manuscript, which was drafted by AV. In addition, EP and MR were in charge of the measures and analyses of meat characteristics, MH and OP of the clinical inspection and selection of the study animals on-farm and AV and CM of the sampling and analysing of saliva cortisol. RP was in charge of sampling and analysing the HSP70 as well as serum cortisol and blood lactate. All authors read and approved the final manuscript.
